# High codon adaptation in citrus tristeza virus to its citrus host

**DOI:** 10.1186/1743-422X-9-113

**Published:** 2012-06-14

**Authors:** Xiao-fei Cheng, Xiao-yun Wu, Hui-zhong Wang, Yu-qiang Sun, Yong-sheng Qian, Lu Luo

**Affiliations:** 1 College of Life and Environmental Science, Hangzhou Normal University, Hangzhou, 310036, China; 2 College of Agricultural and Food Science, Zhejiang Agricultural and Forestry University, Linan, 311300, China

**Keywords:** Citrus tristeza virus, Synonymous codon usage, Citrus sinensis, Codon resemblance, Virus-host interaction

## Abstract

**Background:**

Citrus tristeza virus (CTV), a member of the genus *Closterovirus* within the family *Closteroviridae*, is the causal agent of citrus tristeza disease. Previous studies revealed that the negative selection, RNA recombination and gene flow were the most important forces that drove CTV evolution. However, the CTV codon usage was not studied and thus its role in CTV evolution remains unknown.

**Results:**

A detailed comparative analysis of CTV codon usage pattern was done in this study. Results of the study show that although in general CTV does not have a high degree of codon usage bias, the codon usage of CTV has a high level of resemblance to its host codon usage. In addition, our data indicate that the codon usage resemblance is only observed for the woody plant-infecting closteroviruses but not the closteroviruses infecting the herbaceous host plants, suggesting the existence of different virus-host interactions between the herbaceous plant-infecting and woody plant-infecting closteroviruses.

**Conclusion:**

Based on the results, we suggest that in addition to RNA recombination, negative selection and gene flow, host plant codon usage selection can also affect CTV evolution.

## Background

Protein synthesis takes place when genetic codes stored in the genome is translated at ribosomes in a three-nucleotide manner from the 5' to the 3' end. Each three-nucleotides represents a unique genetic codon for an amino acid or as a translation stop codon. There are 64 codons for the 20 standard amino acids and three stop codons, resulting in more than one codon for most of the 20 amino acids. Codons encode the same amino acid are known as synonymous codons. The synonymous codons are not used in the same frequency in different genes or organisms, indicating the existence of biases in codon usage [1]. Bias in codon usage may play an important role in evolution history of genes or organisms [2]. It was reported that the codon usage bias can be influenced by many factors including translation selection, mutation pressure, gene transfer, amino acid conservation, RNA stability, hypersaline adaption and growth conditions [3-5]. Among these factors, mutation pressure and translation selection were thought to be the key factors shaping the codon usage bias [6].

Viruses are obligate intracellular parasites which dependent on host cells for their genome replication and protein synthesis. It was reported that viral codon usage bias is determined by both virus itself and its host. Similar to other organisms, both mutation pressure and translation selection play a key role in shaping viral codon usage bias [7-10]. Other factors that affect viral codon usage bias include fine-tuning translation kinetic selection [11,12], codon pair bias [13], and escape from cellular antiviral responses through a mechanism involving reduction of CpG dinucleotide [14]. Studies of viral codon usage bias can improve our knowledge not only on virus evolution but also specific interactions between a virus and its host. The codon usage pattern of animal viruses, including human immunodeficiency virus type 1 and hepatitis A virus, has been studied extensively [11,15-19]. For plant viruses this type of study is still rare [8,20,21].

Citrus tristeza virus (CTV), the causal agent of citrus tristeza disease, is a notorious plant RNA virus. CTV causes tremendous economic losses to the citrus industries worldwide [22]. CTV is a non-enveloped, single-stranded positive-sense RNA virus belonging to the genus *Closterovirus* in the family of *Closteroviridae*[23]. Genome RNA (gRNA) of CTV is approximately 19.3 kb in length and contains 12 open reading frames (ORFs) that from the 5' to the 3' end are *ORF1aORF1bp33p6p65p61p27* (encodes the minor coat protein), *p25* (encodes the major coat protein), *p18p13p20*, and *p23*. The 12 ORFs are finally translated into at least 19 different proteins [24]. *ORF1a* and *ORF1b* are translated directly from the gRNA and encode proteins that are required for CTV replication. The ORFs, present on 3′-coterminal subgenomic RNAs, encode proteins that are necessary for CTV replication (*e.g.* p65 and p61), virion assembly (p65, p61, p27 and p25) [25], virus movement (p65, p61, p6, p20) [26], symptom development and asymmetrical accumulation of positive and negative strand viral RNAs during CTV infection (p23) [27-29], and suppression of RNA silencing (p25, p20 and p23) [30]. Functions of CTV p33, p18 and p13 proteins have not been determined.

Isolates of CTV can cause different disease symptoms (*i.e.* yellowing canopies, declining and stunting of trees, and stem pitting) on different indicator citrus plants, indicating the existence of a highly diversified genetic population of CTV in nature [31]. Previous phylogenetic and genetic marker analyses showed that CTV is consists of several genetically distinct genotypes [32,33]. Previous studies also showed that RNA recombination, negative selection and gene flow are the important forces that drive evolution of CTV [34-38]. However, the contribution of codon usage bias to CTV evolution remains unclear. In this study, a detailed comparative analysis was performed using the coding regions for all CTV proteins (refer to full coding region thereafter) to determine the CTV codon usage pattern. Our results show that CTV has a high level of codon usage resemblance to its citrus host, suggesting that codon usage adaptation may also have an important role during CTV evolution.

## Results

### Nucleotide composition properties of CTV full coding region

The effective number of codons (N_C_) of the 20 selected CTV isolates was determined to generate an overall view of the codon usage patterns. Table 1 shows that the N_C_ values of the 20 selected CTV isolates varied from 51.9 to 54.8, with an average value of 53.0 ± 0.6641. This fining suggests that CTV does not possess an excessive overall codon usage bias and the variation of codon usage bias among CTV isolates is small.

**Table 1:** Nucleotide contents of CTV

**Isolate numbers**	**A%**	**A**_**3**_**%**	**U%**	**U**_**3**_**%**	**C%**	**C**_**3**_**%**	**G%**	**G**_**3**_**%**	**(G + C) %**	**(G + C)**_**3**_**%**	**N**_**C**_
1	26.4	20.3	29.9	36.3	17.2	21.6	25.0	22.1	42.2	43.8	53.0
2	27.0	22.3	29.9	36.3	17.2	21.9	24.4	20.0	41.6	41.8	53.8
3	26.8	21.7	30.1	36.5	17.0	21.6	24.7	20.6	41.7	42.2	52.2
4	26.5	20.4	29.9	36.3	17.1	21.8	24.9	21.9	42.1	43.7	52.9
5	26.6	20.8	30.0	36.7	17.1	21.4	24.9	21.5	41.9	42.9	52.7
6	26.7	21.0	29.9	36.2	17.2	22.1	24.7	21.2	41.9	43.2	54.2
7	26.6	20.4	30.0	36.5	17.3	21.9	24.6	21.6	41.9	43.5	53.0
8	26.6	20.4	30.1	36.7	17.2	21.8	24.6	21.6	41.8	43.4	53.0
9	26.8	21.0	30.2	37.1	16.9	21.1	24.6	21.2	41.6	42.3	52.5
10	26.8	21.2	30.1	36.9	17.1	21.0	24.6	21.2	41.7	42.3	52.6
11	26.7	21.0	30.0	36.5	17.0	21.6	24.7	21.4	41.8	42.9	52.9
12	26.9	22.1	30.2	36.8	17.0	21.6	24.3	19.9	41.4	41.5	51.9
13	26.6	20.8	30.1	36.6	17.3	22.1	24.5	20.9	41.8	43.0	54.8
14	26.5	20.9	30.3	36.9	17.2	21.7	24.5	20.9	41.7	42.7	53.6
15	26.4	20.9	30.3	36.9	17.1	21.7	24.6	20.9	41.7	42.6	53.5
16	26.5	20.8	30.3	36.8	17.2	21.9	24.5	20.9	41.7	42.8	53.6
17	26.5	21.0	30.4	37.1	17.0	21.5	24.5	20.8	41.5	42.3	53.4
18	26.4	20.7	30.2	36.4	17.4	22.4	24.4	20.8	41.8	43.2	52.8
19	26.5	20.7	29.9	35.9	17.4	22.7	24.7	21.1	42.1	43.8	52.6
20	26.5	20.7	30.0	36.0	17.4	22.7	24.6	21.1	42.0	43.8	52.6
Average	26.6	20.9	30.1	36.5	17.2	21.9	24.6	21.1	41.8	43.0	53.0

**Figure 1:**
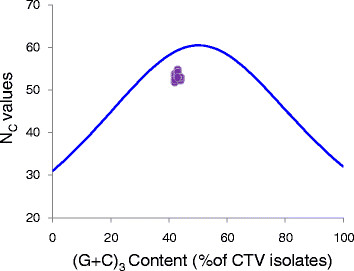
**N**_**C**_**-plot of N**_**C**_**values *versus *(G + C)**_**3 **_**contents of CTV isolates.** Blue curve indicates the expected curve when all codons are used randomly (no selection) and is calculated using the formula reported by Wright previously [39].

The nucleotide abundance was then calculated as another indicator of codon usage bias for CTV (Table 1). The overall Guanine and Cytimidine (G + C) contents in the CTV full coding region and at the synonymous sites (G + C)_3_ fluctuate ranging from 41.4 to 42.2% with an average at 41.8 ± 0.21 and from 41.5 to 43.8% with an average at 43.0 ± 0.68, respectively (Table 1). These results indicate that variation of (G + C) content among CTV isolates in the full coding region and at synonymous sites is small. Comparing the A, U, G and C contents at the synonymous sites (abbreviated as A_3_, U_3_, G_3_ and C_3_), it is clear that the U_3_ value is the highest, ranging from 35.9 to 37.1% with an average at 36.5 ± 0.36. Thus the major codons used by CTV are U-ended. Further comparison of the U, C, G and A contents with the U_3_ C_3_, G_3_, and A_3_ contents indicated that the U and C contents were significantly enriched at the synonymous sites, whereas the G and A were significantly decreased at these synonymous sites (*t* test, *P* < 0.001). To generate a visual display of the main features of codon usage pattern as reported previously by Wright [39], we performed the N_C_-plot, a plot showing N_C_*vs.* (G + C)_3_. In this N_C_-plot (Figure 1), all the CTV isolates clustered together and deviated slightly from the expected curve, which represents the expected codon usage when G + C compositional constraints alone account for the codon usage bias [39]. Our finding implies that CTV is subjected to G + C compositional constraints.

To further confirm this conclusion, we analyzed the cumulative relative synonymous codon usage (RSCU) values for the 20 selected CTV isolates with a total number of 123,535 synonymous codons (Table 2). For amino acids (except Leu) that have more than two synonymous codons (*e.g.* Val, Ser, Pro, Thr, Gly, Arg, Ala and Ile), the codons with the highest RSCU values are all ended with U. For amino acids that have two synonymous codons and are ended with U or C (*e.g.* Phe, His, Asn, Asp, Cys and Tyr), only Tyr displayed a weak preference to codons ended with C (UAC). The RSCU values for amino acids that have two synonymous codons and are ended with A or G (*e.g.* Gln, Lys and Glu) are similar, indicating that a similar codon usage frequency (Table 2). These results demonstrate that CTV likely prefers a U-ended codon usage.

**Table 2:** **Relative synonymous codon usage (RSCU) values in the full coding region of CTV and*Citrus sinensis***

**AA**^**a**^	**Codon**	**N**^**b**^	**CTV**^**c**^	**CS**^**d**^	**AA**	**Codon**	**N**	**CTV**	**CS**
Phe	UUU	4888	1.26^e^	1.05	Gln	CAA	1603	1.24	1.06
	UUC	2888	0.74	0.95		CAG	986	0.76	0.94
Leu	UUA	3748	1.57	0.77	His	CAU	1535	1.03	1.08
	UUG	4825	2.02	1.40		CAC	1453	0.97	0.92
	CUU	2149	0.90	1.58	Asn	AAU	2997	1.00	1.07
	CUC	1216	0.51	0.91		AAC	2992	1.00	0.93
	CUA	1053	0.44	0.53	Lys	AAA	3604	0.94	0.86
	CUG	1354	0.57	0.80		AAG	4077	1.06	1.14
Val	GUU	5171	1.52	1.61	Asp	GAU	4988	1.10	1.29
	GUC	2302	0.68	0.67		GAC	4101	0.90	0.71
	GUA	1861	0.55	0.48	Glu	GAA	4188	1.12	0.95
	GUG	4277	1.26	1.24		GAG	3262	0.88	1.05
Ser	UCU	3742	1.50	1.38	Arg	AGA	2080	1.13	1.82
	UCC	2014	0.81	0.77		AGG	2032	1.11	1.82
	UCA	1642	0.66	1.33		CGU	2814	1.53	0.68
	UCG	3125	1.25	0.71		CGC	1599	0.87	0.56
	AGU	2791	1.12	0.87		CGA	1343	0.73	0.58
	AGC	1642	0.66	0.93		CGG	1136	0.62	0.54
Pro	CCU	2261	1.62	1.31	Cys	UGU	2235	1.24	0.98
	CCC	984	0.71	0.87		UGC	1356	0.76	1.02
	CCA	845	0.61	1.25	Tyr	UAU	2500	0.89	1.05
	CCG	1476	1.06	0.57		UAC	3107	1.11	0.95
Thr	ACU	3455	1.74	1.45	Ala	GCU	3824	1.68	1.58
	ACC	1676	0.84	0.83		GCC	1558	0.68	0.86
	ACA	891	0.45	1.18		GCA	1373	0.60	1.11
	ACG	1933	0.97	0.53		GCG	2373	1.04	0.45
Gly	GGU	4337	1.99	1.13	Ile	AUU	2562	1.21	1.37
	GGC	1311	0.60	0.99		AUC	1681	0.79	0.93
	GGA	1329	0.61	1.07		AUA	2135	1.00	0.70
	GGG	1759	0.81	0.81					

^a^AA is the abbreviation of amino acid.

^b^N, the total numbers for each codon used by the 20 CTV isolates.

^c^CTV, the mean RSCU values of CTV.

^d^CS, the mean RSCU values of *Citrus sinensis*.

^e^The Preferred codons are under lined. A preferred codon is defined by the codon with the highest RSCU value among all available synonymous codons for a certain amino acid. However a codon with the highest RSCU value but lower than 1.1 cannot be defined as the preferred codon, since this value is statistically insignificantly under 95% confidence interval.

### Codon usage patterns of CTV and its host, *citrus sinensis*

**Figure 2:**
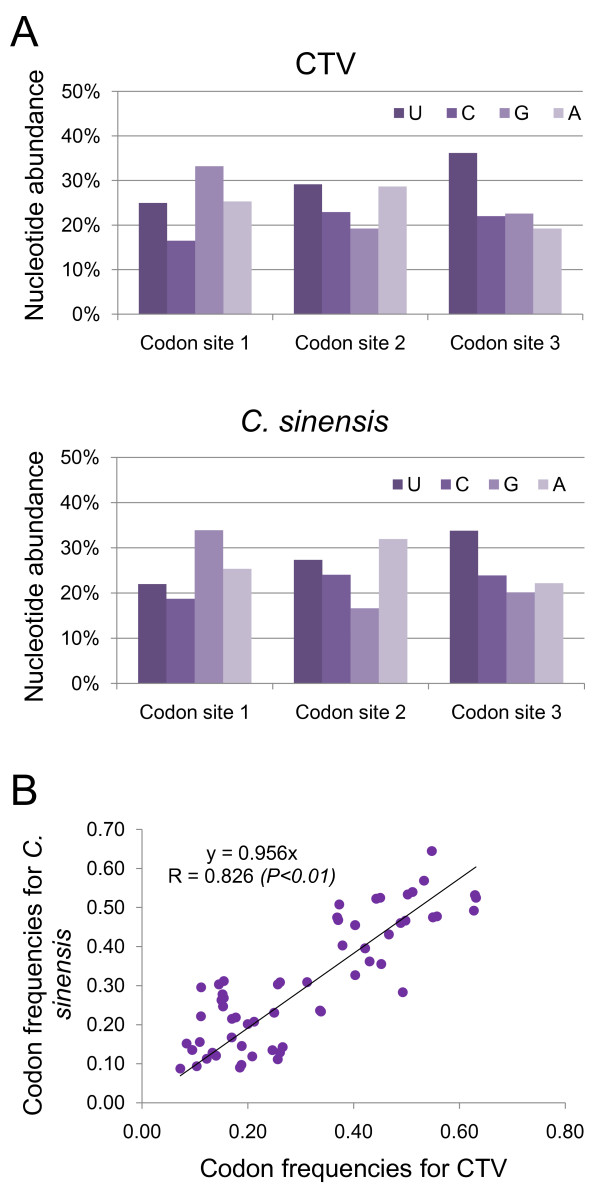
**Comparative analysis of nucleotide composition and codon abundance of CTV and*C. sinensis*. (A)** Frequencies of 4 nucleotides at the three positions within a codon for the full CTV coding region (up panel) and coding region of *C. sinensis* (lower panel). **(B)** Correlation of the codon abundances between CTV and *C. sinensis.*

To compare the codon usage patterns of CTV and its host, we downloaded the codon usage pattern of *C. sinensis* from the Codon Usage Database (http://www.kazusa.or.jp/codon/). Interestingly, our analysis shows that most of the *C. sinensis* preferred codons are also U-ended (Table 2). We then calculated the codon nucleotide abundance for *C. sinensis* and compared it with that of CTV. It was reported previously that for synonymous codons, the second nucleotide site has the strongest constraint, followed by the first nucleotide site [40]. As shown in Figure 2A, CTV has a almost identical nucleotide abundance at the second nucleotide site compared with that of *C. sinensis*. At the first nucleotide site, a similar trend is also evident with slight variations between the two species. At the third nucleotide site, however, both CTV and *C. sinensis* showed a high content of U, indicating that U is preferred by both CTV and *C. sinensis* at the synonymous sites. Interestingly, the second abundant nucleotide at the synonymous sites for *C. sinensis* is C, which is found to be over-represented at the CTV synonymous sites (Table 1). Furthermore, the observed codon usage frequencies for CTV is highly correlated with that for *C. sinensis* (*R* = 0.826, *P < 0.01*) (Figure 2B), indicating that the codon usage of CTV has a high level of resemblance to that of *C. sinensis*.

### Codon usage variations among CTV genotypes

**Figure 3:**
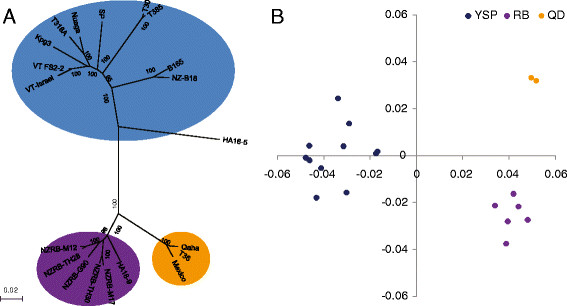
**Codon usage variations of CTV genotypes. (A)** NJ phylogenetic tree of CTV constructed using the entire coding region. The yellowing and stem-pitting CTV group is colored in blue, the trifoliate orange resistance breaking CTV group is colored in purple, and the quick declining CTV group is colored in yellow. **(B)** Distributions of CTV codon usage variation along the first and second axes based on the COA analysis. Coordinates of the three CTV groups are colored in the same way as that shown in Figure 3A.

CTV is known to have several distinct biological genotypes [31-33]. To determine the codon usage variations for these CTV genotypes, a phylogenetic tree was constructed using the full coding region of CTV. Similar to the phylogenetic tree constructed using the CTV full length genomic sequences [33], the yellowing and stem pitting isolates were clustered in the same group (group1), the quick declining isolates were clustered in the group2, and isolates that are capable of breaking CTV resistance in trifoliate orange (*Poncirus trifoliata*) were clustered in the group3 (Figure 3A). To determine the variation of codon usage among the CTV genotypes we conducted a correspondence analysis (COA), a method used to detect major trends in codon usage variations between genes or organisms [41], based on the RSCU values from the 20 selected CTV isolates. Results of the COA extract two major axes. The Axis 1 can explain 37.98% and the Axis 2 can account for 17.18% of the total variations observed. A plot of the two major axes was shown in Figure 3B. In the plot, the three phylogenetic distinct groups are clustered in three independent fields, indicating that these three CTV groups have different trends in codon usage.

A correlation analysis was performed using the nucleotide compositions at the synonymous sites and the two major axes obtained from the COA analysis (Table 3). This analysis allows us to identify the contents that are responsible for the variations [19,42]. Results of the analysis show that only C_3_ has a clear correlation with the two major axes. This indicates that although U is the most preferred nucleotide at the synonymous sites the codon usage variations found among the CTV genotypes were determined by the content of C at the synonymous sites.

**Table 3:** Analysis of correlation between the first two principle axes and nucleotide compositions

**Nucleotide contents**	**Axis 1**	**Axis 2**
A_3_	−0.109	−0.380
U_3_	0.022	−0.589^**^
G_3_	−0.356	0.299
C_3_	0.539^**^	0.504^*^

^**^Correlation is significant at the 0.01 level (2-tailed).

^*^Correlation is significant at the 0.05 level (2-tailed).

### Codon usage adaptation of closteroviruses

**Figure 4:**
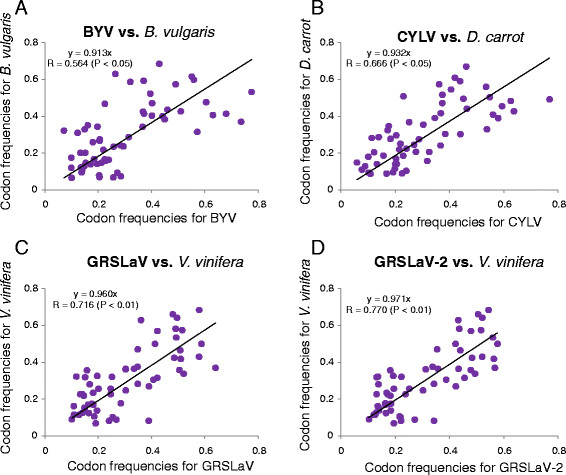
**Correlations of the codon abundances of closteroviruses and their respective host species. (A)** Beet yellows virus (BYV) *versus Beta vulgaris* (beet); **(B)** Carrot yellow leaf virus (CYLV) *versus Daucus carrot* (carrot); **(C)** Grapevine rootstock stem lesion associated virus (GRSLaV) *versus Vitis vinifera* (grapevine); (D) Grapevine leafroll-associated virus 2 (GRLaV-2) *versus V. vinifera*. The codon usage patterns of beet, carrot and grapevine were downloaded from the Codon Usage Database (http://www.kazusa.or.jp/codon/).

The high degree of CTV codon usage adaptation to its host suggests that the adaptation may be a common phenomenon between closteroviruses and their hosts. To confirm this hypothesis, the full length genome sequences of beet yellows virus (AF056575, BYV), carrot yellow leaf virus (NC_013007, CYLV), grapevine rootstock stem lesion associated virus (NC_004724, GRSLaV) and grapevine leafroll-associated virus 2 (NC_007448, GRSLaV-2) were downloaded from the GenBank. The empirical codon frequency of each virus was calculated and compared with that of its host plant: *Beta vulgaris* (beet) for BYV, *Daucus carrot* (carrot) for CYLV, and *Vitis vinifera* (grapevine) for GRSLaV and GRSLaV-2. Results shown in Figure 4 indicate that significant correlation (*P < 0.01*) is observed between grapevine and its two viruses (GRSLaV and GRSLaV-2) but not between beet and BYV or carrot and CYLV. This finding shows that codon usage adaptation to a host is not a common phenomenon of closteroviruses. It occurs only in some closteroviruses.

## Discussion

In this study, a detailed comparative analysis was done to determine CTV codon usage bias. Our results show that in general CTV does not have a high degree of codon usage bias (average N_C_ = 53.0, Table 1), and mutational bias is likely to be the major force that drives CTV codon usage bias (Figure 1). This finding supports the previous reports that mutational bias is the major force that affects the viral codon usage in other viruses [7,8]. However, the deviation of the coordinates from the expected curve shown in the N_C_-plot cannot be simply explained by the mutational bias as suggested by Wright previously [39]. It is possible that this deviation is caused by either the G/C-biased mutation pressure or the negative/positive selection of codons ended with C and/or G as described before [39]. In deed, comparing the A, U, G, and C contents in the full coding region with that found at the synonymous codon sites, C is over-represented at the synonymous codon sites in addition to U (Table 1). Interestingly, analysis of selective pressure that act on different codons suggested that the full coding region of CTV is subjected mostly to the purifying selection described by Martin et al. [35]. It is possible that the enrichment of C at the synonymous sites is caused by negative selection other than the C biased mutational pressure. Furthermore, results of COA show that the C content at the synonymous sites is the major factor that determines the codon usage variation among the CTV genotypes (Table 3). Because different CTV genotypes were reported to have different host origins [43], the enrichment of C at the synonymous sites is likely caused by the selection of the host.

Our results also show that codon usage of CTV has a high level of resemblance to that of its citrus host. This is because i) both CTV and citrus have significantly higher content of U at the synonymous codon sites; ii) most of the preferred codons in CTV and citrus are the same; iii) a high correlation exists in codon frequencies between CTV and citrus. This result is understandable when consider the specific relationship between CTV and its host. CTV is restricted to citrus and it is generally accepted that the virus co-evolved with the host species [44]. Whereas, citrus is a woody plant and can grow in field for hundreds of years [22]. After successful infection, the virus can survive in this host for a very long period of time. This long term infection gives CTV an opportunity to select and adapt optional codons generated during virus replication. As discussed above, the C_3_ content is the major factor that determines the codon usage variation among the CTV genotypes. Our data also indicate that the degrees of codon usage adaptations by different CTV genotypes to *C. sinensis* are different, suggesting that the codon usage variation may reflect specific interactions between the CTV genotypes and their original hosts. Because detailed genetic information on CTV original citrus hosts are missing, we are unable confirm the codon usage adaptation by CTV genotypes to their respective hosts. Nevertheless, our results presented in this paper show that CTV and citrus is an idea model for studies of virus and host coevolution.

Bahir et al. suggested previously that adaptation of codon usage varied among different viral genes and the highest degree of adaptation was observed for genes that expressed to high levels in cells, such as the viral CP [21]. In this study we also tried to analysis the variations of codon usage among CTV genes, and the different host effects on these genes. However, this attempt was un-succeeded because the number of codons used by some CTV genes are limited and thus many synonymous codons may not be observed. This may cause artificial errors when compare virus codon usage frequency with that of its host.

High adaptation of codon usage was previously reported for several viruses including those belonging to the family *Flaviviridae*, and bacteria-infecting and human viruses [14,21]. We proposed that high codon adaptation phenomenon might exist in all viruses in the genus *Closterovirus* since the codon usage patterns of different closteroviruses are highly resemblance to each other (data not shown). However, our results show that the high degree of codon resemblance is only observed between the woody plant-infecting closteroviruses and their woody hosts, but not the herbaceous plants-infecting closteroviruses and their herbaceous hosts (Figure 4). This difference may be caused partially by the different longevity of closteroviruses in their infected herbaceous or woody plants. It is known that the woody plant-infecting closteroviruses can exist in their host plants for a very long period of time. In addition, all woody plant-infecting closteroviruses infect only a few closely related species within the same genus. This narrow host range feature may also have a role in this unusual high codon adaptation phenomenon. For example, the natural hosts of CTV are limited only to a few species within the genus of *Citrus*[22].

## Conclusion

A detailed comparative analysis of CTV codon usage pattern was performed in this study. Results of the study show that the overall codon usage of CTV is highly resemble that of its host, *C. sinensis*. Our results also show that the codon usage resemblance is only observed for the woody plant-infecting closteroviruses but not the closteroviruses infecting the herbaceous host plants. This observation implies the existence of different virus-host interactions between the herbaceous plant-infecting and woody plant-infecting closteroviruses. In conclusion, our results indicate that in addition to RNA recombination, negative selection and gene flow, host codon usage selection can also have an important role in CTV evolution.

## Materials and methods

### Source of sequence data

Full length genome sequences of CTV, BYV, CYLV, GRSLaV, and GRSLaV-2 were downloaded from the GenBank (http://www.ncbi.nlm.nih.gov/). To establish a sequence data set for CTV, isolates share less than 98% sequence identity were downloaded and the final data set consists of 20 CTV isolates (Table 4). The accession numbers and other information on these isolates are listed in Table 4. For codon usage analysis open reading frames (ORFs) with less than 150 nucleotides were excluded as described before [45].

**Table 4:** The information of 20 CTV isolates used in this study

**Isolate numbers**	**Strain name**	**length (nt)**^**a**^	**Biological property**	**Accession No.**
1	B165	18585	YSP^b^	EU076703
2	kpg3	18555	YSP	HM573451
3	HA16-5	18567	YSP	GQ454870
4	NZ-B18	18498	YSP	FJ525436
5	SP	18498	YSP	EU857538
6	T318A	18576	YSP	DQ151548
7	T30	18495	YSP	AF260651
8	T385	18495	YSP	Y18420
9	VT-FS2-2	18549	YSP	EU937519
10	VT-Israel	18474	YSP	U56902
11	Nuaga	18549	YSP	AB046398
12	HA18-9	18549	RB^c^	GQ454869
13	NZRB-G90	18498	RB	FJ525432
14	NZRB-TH28	18498	RB	FJ525433
15	NZRB-TH30	18513	RB	FJ525434
16	NZRB-M12	18498	RB	FJ525431
17	NZRB-M17	18516	RB	FJ525435
18	Mexico	18516	QD^d^	DQ272579
19	Qaha	18588	QD	AY340974
20	T36	18588	QD	NC_001661

^a^non-coding regions were excluded.

^b^YSP, yellowing and stem pitting.

^c^RB, resistance breaking.

^d^QD, quick declining.

The codon usage pattern of *C. sinensisB. vulgarisD. carrot*, and *V. vinifera* were downloaded from the Condon Usage Database (http://www.kazusa.or.jp/codon/), which were tabulated based on all available sequences in the international DNA sequence databases [46].

### Phylogenetic analysis

Phylogenetic tree was constructed using the Neighbor-joining (NJ) method described in the MEGA 5.0 software [47]. The nucleotide substitution model, mutation rate and mutation pattern were determined using the Model Selection Function described also in the MEGA 5.0 software. The Bootstrapped confidence interval is based on 1000 replicates.

### Composition analysis of full coding regions of CTV isolates

Analysis of compositional properties of all CTV ORFs, including (G + C), (G + C)_3_, A_3_, U_3_, G_3_ and C_3_, was performed using the CodonW version 1.4.2 (John Peden, available at http://codonw.sourceforge.net/index.html). The nucleotide contents at the first and second codon positions were calculated as described by Wang et al. previously [48].

### Measurement of effective number of codons

Effective number of codons (N_C_) has been used as a measurement for synonymous codon usage bias in genes and is considered to be independent of the gene length and amino acid composition [39]. The N_C_ value ranging from 20 to 61 is often used to determine the degree of codon usage bias in a gene [39]. For example, a gene with a N_C_ value at or below 35 is considered to have a strong codon usage bias, whereas a gene with a N_C_ value of 61 indicates that all available codons are used equally [39]. In this study the N_C_ values were calculated using the CodonW version 1.4.2.

### Measurement of relative synonymous codon usage (RSCU)

RSCU value is the ratio of observed to expected frequency of a codon and reflects the bias of synonymous codon usage without the influence of amino acid composition and the abundance of synonymous codons [49]. A RSCU value above 1.0 indicates a positive codon usage bias, a value below 1.0 implies a negative codon usage bias, and a value at 1.0 indicates no codon usage bias for the synonymous codons [49]. In this study the RSCU value is calculated using the General Codon Usage Analysis (GCUA) software available at http://bioinf.may.ie/GCUA/calculatecodon.html[50].

### Correspondence analysis (COA) of synonymous codon usage

COA is a commonly used multivariate statistical analysis method [51] and has been used to investigate the major trends in codon usage variation between genes or organisms [19,41,42]. In this study, COA is used to analyze codon usage variations between CTV isolates. In the analysis, the RSCU values of synonymous codons (excluding Met, Trp and the three termination codons) were treated as 59 dimensional vectors. Therefore, each CTV isolate can be represented by a 59 coordinates (RSCU values). The calculation was done using the CodonW 1.4.2 software.

### Correlation analysis

Correlation analysis was performed to determine the relationship between nucleotide composition and synonymous codon usage pattern using the Spearman’s rank correlation analysis described in the SPSS 16.0 software (SPSS Lnc., USA).

## Abbreviations

CTV, Citrus tristeza virus; ORF, Open reading frame; NC, Effective number of codon; RSCU, Relative synonymous codon usage; COA, Correspondence analysis.

## Competing interests

The authors declare no competing interests.

## Authors’ contributions

XC involved in data calculation, results analysis and manuscript preparation; XW involved in data collection, results analysis and manuscript revision; HW and YS involved in data analysis and manuscript preparation; YQ and LL involved in data visualization; All authors have read and approved the final submission of the manuscript.

## References

[B1] Grantham R Gautier C Gouy M Mercier R Pave A Codon catalog usage and the genome hypothesis Nucl Acids Res 1980 8 14 10.1093/nar/8.1.197-c PMC327256 6986610

[B2] Ingvarsson PK Molecular evolution of synonymous codon usage in Populus BMC Evol Biol 2008 8 307 10.1186/1471-2148-8-307 18983655 PMC2586637

[B3] Ermolaeva MD Synonymous codon usage in bacteria Curr Issues Mol Biol 2001 3 91 97 11719972

[B4] Lynn DJ Singer GA Hickey DA Synonymous codon usage is subject to selection in thermophilic bacteria Nucl Acids Res 2002 30 4272 4277 10.1093/nar/gkf546 12364606 PMC140546

[B5] Paul S Bag S Das S Harvill E Dutta C Molecular signature of hypersaline adaptation: insights from genome and proteome composition of halophilic prokaryotes Genome Biol 2008 9 R70 10.1186/gb-2008-9-4-r70 18397532 PMC2643941

[B6] Sharp PM Stenico M Peden JF Lloyd AT Codon usage: mutational bias, translational selection, or both? Biochem Soc Trans 1993 21 835 841 8132077 10.1042/bst0210835

[B7] Jenkins GM Holmes EC The extent of codon usage bias in human RNA viruses and its evolutionary origin Virus Res 2003 92 1 7 10.1016/S0168-1702(02)00309-X 12606071

[B8] Adams MJ Antoniw JF Codon usage bias amongst plant viruses Arch Virol 2004 149 113 135 14689279 10.1007/s00705-003-0186-6

[B9] Zhou J Liu WJ Peng SW Sun XY Frazer I Papillomavirus capsid protein expression level depends on the match between codon usage and tRNA availability J Virol 1999 73 4972 4982 10233959 10.1128/jvi.73.6.4972-4982.1999 PMC112541

[B10] Karlin S Blaisdell BE Schachtel GA Contrasts in codon usage of latent versus productive genes of epstein-barr virus: data and hypotheses J Virol 1990 64 4264 4273 2166815 10.1128/jvi.64.9.4264-4273.1990 PMC247892

[B11] Aragonès L Guix S Ribes E Bosch A Pintó RM Fine-tuning translation kinetics selection as the driving force of codon usage bias in the hepatitis A virus capsid PLoS Pathog 2010 6 e1000797 10.1371/journal.ppat.1000797 20221432 PMC2832697

[B12] Aragonès L Bosch A Pintó RM Hepatitis A virus mutant spectra under the selective pressure of monoclonal antibodies: codon usage constraints limit capsid variability J Virol 2008 82 1688 1700 10.1128/JVI.01842-07 18057242 PMC2258700

[B13] Coleman JR Papamichail D Skiena S Futcher B Wimmer E Mueller S Virus attenuation by genome-scale changes in codon pair bias Science 2008 320 1784 1787 10.1126/science.1155761 18583614 PMC2754401

[B14] Lobo FP Mota BEF Pena SDJ Azevedo V Macedo AM Tauch A Machado CR Franco GR Virus-host coevolution: common patterns of nucleotide motif usage in Flaviviridae and their hosts PLoS One 2009 4 e6282 10.1371/journal.pone.0006282 19617912 PMC2707012

[B15] Sharp PM What can AIDS virus codon usage tell us? Nature 1986 324 114 10.1038/324114a0 3641061

[B16] Meintjes PL Rodrigo AG Evolution of relative synonymous codon usage in human immunodeficiency virus type-1 J Bioinform Comput Biol 2005 3 157 168 10.1142/S0219720005000953 15751118

[B17] Pintó RM Aragonès L Costafreda MI Ribes E Bosch A Codon usage and replicative strategies of hepatitis A virus Virus Res 2007 127 158 163 10.1016/j.virusres.2007.04.010 17524513

[B18] D' Andrea L Pinto RM Bosch A Musto H Cristina J A detailed comparative analysis on the overall codon usage patterns in hepatitis A virus Virus Res 2011 157 19 24 10.1016/j.virusres.2011.01.012 21296111 PMC7172775

[B19] Zhang Y Liu Y Liu W Zhou J Chen H Wang Y Ma L Ding Y Zhang J Analysis of synonymous codon usage in hepatitis A virus Virol J 2011 8 174 10.1186/1743-422X-8-174 21496278 PMC3087699

[B20] Xu XZ Liu QP Fan LJ Cui XF Zhou XP Analysis of synonymous codon usage and evolution of begomoviruses J Zhejiang Univ Sci B 2008 9 667 674 10.1631/jzus.B0820005 18763298 PMC2528880

[B21] Bahir I Fromer M Prat Y Linial M Viral adaptation to host: a proteome-based analysis of codon usage and amino acid preferences Mol Syst Biol 2009 5 311 19888206 10.1038/msb.2009.71 PMC2779085

[B22] Moreno P Ambrós S Albiach-Mart MR Guerri J Peña L Citrus tristeza virus: a pathogen that changed the course of the citrus industry Mol Plant Pathol 2008 9 251 268 10.1111/j.1364-3703.2007.00455.x 18705856 PMC6640355

[B23] Martelli GP Agranovsky AA Bar-Joseph M Boscia D Candresse T Coutts RH Dolja V Hu J Jelkmann W Karasev AV Andrew MQK, Elliot L, Michael JA, Eric B, CarstensA2 - Andrew MQ, King ELMJA, Eric BC Closteroviridae Virus Taxonomy 2012 San Diego: Elsevier 987 1001

[B24] Karasev AV Boyko VP Gowda S Nikolaeva OV Hilf ME Koonin EV Niblett CL Cline K Gumpf DJ Lee RF Complete sequence of the citrus tristeza virus RNA genome Virology 1995 208 511 520 10.1006/viro.1995.1182 7747424

[B25] Satyanarayana T Gowda S Mawassi M Albiach-Marti MR Ayllon MA Robertson C Garnsey SM Dawson WO Closterovirus encoded HSP70 homolog and p61 in addition to both coat proteins function in efficient virion assembly Virology 2000 278 253 265 10.1006/viro.2000.0638 11112500

[B26] Tatineni S Robertson CJ Garnsey SM Bar-Joseph M Gowda S Dawson WO Three genes of citrus tristeza virus are dispensable for infection and movement throughout some varieties of citrus trees Virology 2008 376 297 307 10.1016/j.virol.2007.12.038 18456299

[B27] Satyanarayana T Gowda S Ayllon MA Albiach-Marti MR Rabindran S Dawson WO The p23 protein of citrus tristeza virus controls asymmetrical RNA accumulation J Virol 2002 76 473 483 10.1128/JVI.76.2.473-483.2002 11752137 PMC136848

[B28] Fagoaga C Lopez C Moreno P Navarro L Flores R Pena L Viral-like symptoms induced by the ectopic expression of the p23 gene of citrus tristeza virus are citrus specific and do not correlate with the pathogenicity of the virus strain Mol Plant Microbe Interact 2005 18 435 445 10.1094/MPMI-18-0435 15915642

[B29] Ghorbel R LÓpez C Fagoaga C Moreno P Navarro L Flores R Peña L Transgenic citrus plants expressing the citrus tristeza virus p23 protein exhibit viral-like symptoms Mol Plant Pathol 2001 2 27 36 10.1046/j.1364-3703.2001.00047.x 20572989

[B30] Lu R Folimonov A Shintaku M Li W-X Falk BW Dawson WO Ding S-W Three distinct suppressors of RNA silencing encoded by a 20-kb viral RNA genome Proc Natl Acad Sci U S A 2004 101 15742 15747 10.1073/pnas.0404940101 15505219 PMC524217

[B31] Niblett CL Genc H Cevik B Halbert S Brown L Nolasco G Bonacalza B Manjunath KL Febres VJ Pappu HR Lee RF Progress on strain differentiation of citrus tristeza virus and its application to the epidemiology of citrus tristeza disease Virus Res 2000 71 97 106 10.1016/S0168-1702(00)00191-X 11137165

[B32] Hilf ME Mavrodieva VA Garnsey SM Genetic marker analysis of a global collection of isolates of citrus tristeza virus: characterization and distribution of CTV genotypes and association with symptoms Phytopathology 2005 95 909 917 10.1094/PHYTO-95-0909 18944413

[B33] Harper SJ Dawson TE Pearson MN Isolates of citrus tristeza virus that overcome Poncirus trifoliata resistance comprise a novel strain Arch Virol 2010 155 471 480 10.1007/s00705-010-0604-5 20352212

[B34] Rubio L Ayllon MA Kong P Fernandez A Polek M Guerri J Moreno P Falk BW Genetic variation of citrus tristeza virus isolates from California and Spain: evidence for mixed infections and recombination J Virol 2001 75 8054 8062 10.1128/JVI.75.17.8054-8062.2001 11483750 PMC115049

[B35] Martin S Sambade A Rubio L Vives MC Moya P Guerri J Elena SF Moreno P Contribution of recombination and selection to molecular evolution of citrus tristeza virus J Gen Virol 2009 90 1527 1538 10.1099/vir.0.008193-0 19264625

[B36] Weng Z Barthelson R Gowda S Hilf ME Dawson WO Galbraith DW Xiong Z Persistent infection and promiscuous recombination of multiple genotypes of an RNA virus within a single host generate extensive diversity PLoS One 2007 2 e917 10.1371/journal.pone.0000917 17878952 PMC1975466

[B37] Vives MC Rubio L Sambade A Mirkov TE Moreno P Guerri J Evidence of multiple recombination events between two RNA sequence variants within a citrus tristeza virus isolate Virology 2005 331 232 237 10.1016/j.virol.2004.10.037 15629767

[B38] Melzer MJ Borth WB Sether DM Ferreira S Gonsalves D Hu JS Genetic diversity and evidence for recent modular recombination in Hawaiian citrus tristeza virus Virus Genes 2010 40 111 118 10.1007/s11262-009-0409-3 19834797

[B39] Wright F The 'effective number of codons' used in a gene Gene 1990 87 23 29 10.1016/0378-1119(90)90491-9 2110097

[B40] Mac Dónaill DA Manktelow M Molecular informatics: quantifying information patterns in the genetic code Mol Simulat 2004 30 267 272 10.1080/08927020310001638749

[B41] Su MW Lin HM Yuan HS Chu WC Categorizing host-dependent RNA viruses by principal component analysis of their codon usage preferences J Comput Biol 2009 16 1539 1547 10.1089/cmb.2009.0046 19958082

[B42] Liu YS Zhou JH Chen HT Ma LN Pejsak Z Ding YZ Zhang J The characteristics of the synonymous codon usage in enterovirus 71 virus and the effects of host on the virus in codon usage pattern Infect Genet Evol 2011 11 1168 1173 10.1016/j.meegid.2011.02.018 21382519 PMC7185409

[B43] Ayllon MA Lopez C Navas-Castillo J Garnsey SM Guerri J Flores R Moreno P Polymorphism of the 5' terminal region of citrus tristeza virus (CTV) RNA: incidence of three sequence types in isolates of different origin and pathogenicity Arch Virol 2001 146 27 40 10.1007/s007050170188 11266215

[B44] Bar-Joseph M Marcus R Lee RF The continuous challenge of ctrus tristeza virus control Ann Rev Phytopathol 1989 27 291 316 10.1146/annurev.py.27.090189.001451

[B45] Das S Paul S Dutta C Synonymous codon usage in adenoviruses: influence of mutation, selection and protein hydropathy Virus Res 2006 117 227 236 10.1016/j.virusres.2005.10.007 16307819

[B46] Nakamura Y Gojobori T Ikemura T Codon usage tabulated from international DNA sequence databases: status for the year 2000 Nucleic Acids Res 2000 28 292 10.1093/nar/28.1.292 10592250 PMC102460

[B47] Tamura K Peterson D Peterson N Stecher G Nei M Kumar S MEGA5: molecular evolutionary genetics analysis using maximum likelihood, evolutionary distance, and maximum parsimony methods Mol Biol Evol 2011 28 2731 2739 10.1093/molbev/msr121 21546353 PMC3203626

[B48] Wang M Liu YS Zhou JH Chen HT Ma LN Ding YZ Liu WQ Gu YX Zhang J Analysis of codon usage in newcastle disease virus Virus Genes 2011 42 245 253 10.1007/s11262-011-0574-z 21249440 PMC7088932

[B49] Sharp PM Li W-H Codon usage in regulatory genes in Escherichia coli does not reflect selection for ‘rare’ codons Nucl Acids Res 1986 14 7737 7749 10.1093/nar/14.19.7737 3534792 PMC311793

[B50] McInerney JO GCUA: general codon usage analysis Bioinformatics 1998 14 372 373 10.1093/bioinformatics/14.4.372 9632833

[B51] Greenacre MJ Theory and applications of correspondence analysis 1984 London: Academic

